# Adipokines and inflammatory markers in elderly subjects with high risk of type 2 diabetes and cardiovascular disease

**DOI:** 10.1038/s41598-018-31144-8

**Published:** 2018-08-24

**Authors:** Tuula Saukkonen, Shivaprakash Jagalur Mutt, Jari Jokelainen, Anna-Maria Saukkonen, Ghulam Shere Raza, Toni Karhu, Pirjo Härkönen, Jürgen Eckel, Karl-Heinz Herzig, Ulla Rajala, Sirkka Keinänen-Kiukaanniemi

**Affiliations:** 1 Center for Life Course Health Research, University of Oulu, Oulu, Finland; 2 Oulunkaari Ky, Federation of Municipalities, Piisilta 1, Ii, Oulu, Finland; 3 Research Unit of Biomedicine, Department of Physiology & Biocenter of Oulu, Medical Research Center (MRC), University of Oulu, and University Hospital, Oulu, Finland; 4 Unit of General Practice, Oulu University Hospital, Oulu, Finland; 5 Oulu Deaconess Institute/Diapolis Oy Research Unit, Oulu, Finland; 6 Paul-Langerhans-Group for Integrative Physiology, German Diabetes Center (GDC), Düsseldorf, Germany; 7 Department of Gastroenterology and Metabolism, Poznan University of Medical Sciences, Poznań, Poland

## Abstract

Inflammation plays a significant role in pathogenesis of diabetes and atherosclerosis. Increased adiposity with an upregulation of cytokines in prediabetes has been associated with vascular inflammation and considered a leading causal factor for type 2 diabetes (T2D). Information on adipokines and inflammatory markers in prediabetes, defined by hemoglobin A1C (HbA1c) 5.7–6.4% in addition to impaired fasting glucose (IFG) and impaired glucose tolerance (IGT), are sparse. We conducted a population–based cross-sectional study (part of a follow-up study) of inhabitants of Oulu, Finland, born in 1935. Inflammatory markers and traditional risk markers of 367 subjects were measured. The glucose status was determined by an oral glucose tolerance test (OGTT) and HbA1c. Inflammatory markers and glycemic levels were analysed using analysis of covariance (ANCOVA). Of the participants, 193 were normoglycemic, 82 had prediabetes and 40 T2D. Inflammatory cytokines were significantly higher in subjects with prediabetes as compared to normoglycemic subjects: IL-4 (14.9 vs 5.9 pg/ml, p = 0.041), IP-10 (251 vs 209 pg/ml, p = 0.05), TNF-α (10.4 vs 6.9 pg/ml, p = 0.027), RANTES (43.3 vs 33.1 pg/ml, p = 0.009), CD40L (3708 vs 1671 pg/ml, p = 0.010) and VEGF (269 vs 174 pg/ml, p = 0.013). These inflammatory cytokines remained significant even after adjustment for waist circumference. The differences in inflammatory markers in prediabetic and T2D subjects were not statistically significant. Prediabetes was associated with low-grade inflammation with increased inflammatory cytokine levels, while the levels in prediabetic subjects were comparable to those in T2D subjects. The associations were independent of visceral adiposity.

## Introduction

Inflammation plays a significant role in pathogenesis of diabetes^1^ and atherosclerosis^2^. The white visceral adipose tissue in the obese state actively secretes inflammatory mediators termed cytokines. Several of these are thought to play a central role in the pathophysiology of atherogenesis^3^. Tumor necrosis factor α (TNF-α), Interleukin 4 (IL-4), Interferon gamma (INF)-inducible protein 10 (IP-10), soluble CD40 ligand (CD40L) and Regulated upon activation normal T-cell expressed and secreted (RANTES) are considered to be acute-phase reactants of inflammation and have been associated with vascular inflammation and increased the risk of cardiovascular events^3–5^.

Immunomodulatory treatments affect glycemia, β cell function and insulin resistance in patients with type 2 diabetes (T2D)^6,7^. The evaluation of the molecular pathways showed that blocking e.g., TNF-α stimulated c-Jun N terminal kinase (JNK) and/or I kappa beta kinase (I kappa K), alleviates insulin resistance^8^. Anti-inflammatory substances like salicylic acid in addition with other drugs^9^ have been used to treat hyperglycemia^10,11^.

The diagnosis of diabetes is based on glucose levels – either fasting plasma glucose (FPG) ≥7.0 or 2 hr 75 g oral glucose tolerance test (OGTT) ≥11.1 mmol/L. Subjects with elevated fasting blood glucose (FBG) of 5.6–6.9 mmol/L, 2-hour blood glucose level of ≥7.8 and <11.1 mmol/L, or elevated A1C (HbA1c 5.7–6.4%) have been classified as prediabetic by the American Diabetes Association (ADA)^12^. These prediabetic subjects, characterized by an impaired fasting glucose (IFG) and/or impaired glucose tolerance (IGT), have an increased risk to develop T2D^1^, cardiovascular disease (CVD) and an increased all-cause mortality^13^. Prediabetic subjects (PreDM) are very likely to progress to T2D, which could be prevented by interventions – e.g. lifestyle changes.

Information on the inflammatory markers in prediabetic subjects is limited in spite of the fact that their cardiovascular risks are increased. TNF-α has been shown to correlate with the increased glucose levels and blood pressure in prediabetic subjects^3,5^. High levels of IP-10 have been found in diabetic subjects and also in obese adolescents with IGT^14,15^.

As an adipokine, secreted from adipose tissue, dipeptidyl peptidase (DPPIV) levels were elevated in obese subjects and associated with inflammation and T2D^16^. Overall, the current available studies analyzed only small groups and individual cytokines.

We therefore conducted a population-based observational cross sectional study (1) to determine cytokine and adipokine levels in prediabetic subjects, defined by either IFG, IGT and/or HbA1c, (2) to compare the levels of inflammatory markers in prediabetic, T2D and normoglycemic subjects (NGT) and (3) to evaluate whether these inflammatory markers were associated with traditional cardiovascular risk markers.

## Materials and Methods

### Study design

**Figure 1:**
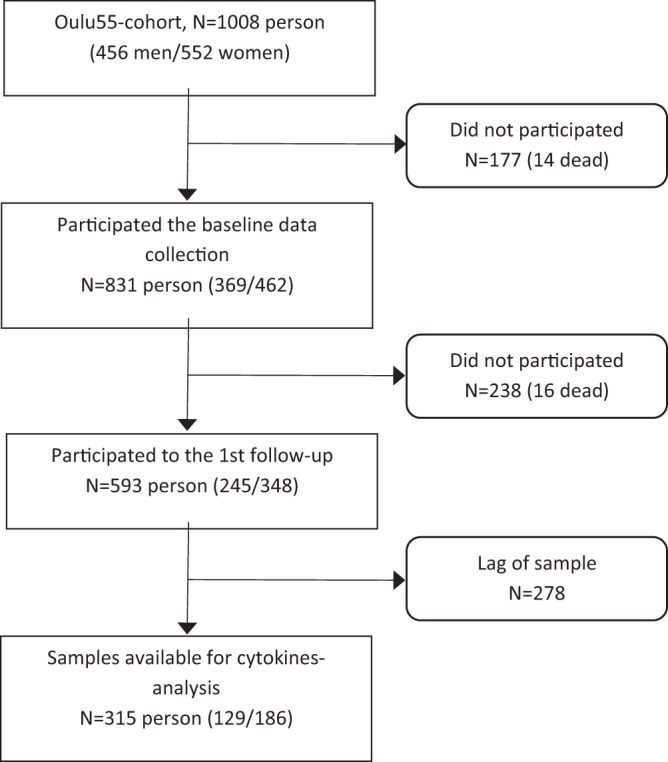
Flow chart of the study.

This cross-sectional study, conducted between 1996 and 1998, was part of a longer follow-up study (started in 1992) to assess the prevalence of T2D and IGT, among inhabitants of the city of Oulu (65^◦^ latitude), Finland, born in 1935. Eight hundred thirty-one (831) inhabitants were invited to participate the second follow-up study, of whom 593 (245 men, 348 women) enrolled. Prediabetes was defined if subjects had either IFG, FBG 5.6–6.9 mmol/L, IGT, 2-hour blood glucose ≥7.8 and <11.1 mmol/L, or elevated HbA1c 5.7–6.4%^12^. Subjects with 2 h glucose ≥11.1 mmol/L, FBG ≥6.1 mmol/L and/or HbA1C (≥6.5%) were diagnosed as T2D. Participants with a known history of T2D were excluded. Inflammatory markers were measured from 315 (53,1%) subjects as shown in flow chart (Fig. 1). The study protocol was approved by the Ethics Committee of the Faculty of Medicine, University of Oulu, Finland and in compliance with the National Guidelines and legislation and the Declaration of Helsinki. All subjects gave their written informed consent. The study protocol of the anthropometric measurements and the clinical data collection has been described in detail previously^17–19^.

### Biochemical measurements

After a 12 h overnight fast, venous blood samples were drawn at 08:00–10:00 am for fasting glucose and HbA1c. A standardized 75-g oral glucose tolerance test (OGTT) was performed and the 2-h glucose levels were determined from capillary blood samples. HbA1c was analyzed with the Bayer DCA2000 Analyzer, calibrated to the Diabetes Control and Complications Trial standard (DCTT). Serum immunoreactive insulin concentration was measured by radioimmunoassay (RIA) using the Phadeseph Insulin RIA100 kit (Pharmacia Diagnostics AB, Uppsala, Sweden). Insulin resistance was calculated using HOMA-IR. Concentrations of whole blood glucose were converted to equivalent plasma glucose concentrations, using a previously described formula^20^.

### Analysis of Inflammatory markers

Inflammatory markers were analyzed using a the Bio-Plex 200 system based on Luminex xMAP technology (Bio-Rad Laboratories Inc., CA, USA) with Milliplex human chemokine/cytokine and CVD/cytokine kits (Cat# HCYTOMAG-60K-12 and Cat# SPR349, Millipore, St Charles, MO, USA) as previously described^21^. Plasma DPPIV levels were measured using the Human DPPIV/CD26 Quantikine ELISA Kit (Cat# DC260, R&D Systems, Inc., UK).

### Statistical analysis

Continuous variables were presented as means and standard deviations and categorical variables as proportions. t-tests were used to compare means between two groups, and for categorical variables the Chi-Square test was used. Triglycerides levels, fasting and 2-hour glucose, HbA1c, fasting insulin and HOMA-IR and inflammation markers were log-transformed to normalize their distributions for analysis. ANCOVA was used to compare the inflammatory markers in normoglycemic subjects and those with prediabetes or T2D, and the analysis adjusted for waist circumference. Associations between inflammation markers, anthropometric and metabolic variables were assessed by partial Spearman correlations coefficients, adjusted for sex.

To correct for multiple testing, the false discovery rate (FDR) was controlled at 0.05 using the Benjamini-Hochberg method^22^. P < 0.05 was considered statistically significant. Statistical analysis was performed using SAS 9.4 TS for Windows (SAS Institute Inc., Gary NC, USA). Visualization of the correlation matrix was performed using R package “corrplot”^23^.

## Results

One hundred ninety-three (193) subject were normoglycemic (NGT), 82 subjects were prediabetic and 40 diagnosed as new T2D.

The anthropometric and clinical characteristics of the 129 men and 186 women in the cohort are shown in Table 1. The waist circumferences of men compared to women were increased (96.3 cm vs. 83.1 cm, p <0.001), with higher HOMA-IR (1.6 vs.1.3, p = 0.001) and lower HDL-cholesterol levels (1.3 vs.1.6 p < 0.001). Men had a higher prevalence of T2D than women (17.3% vs. 9.9%, p = 0.056).

**Table 1:** Characteristics of the subjects (Oulu Cohort 1935) by gender.

	Men	Women	p-value
*Number of subjects*	129	186	
Age	62.1 (0.7)	62.1 (0.6)	
BMI (kg/m^2^)	27.9 (3.5)	27.4 (4.3)	0.278
Waist circumference (cm)	96.3 (9.9)	83.1 (11.1)	<0.001
Systolic BP (mmHg)	142 (16.7)	143 (17.0)	0.633
Diastolic BP (mmHg)	79.0 (7.5)	78.7 (8.1)	0.787
HDL Cholesterol (mmol/l)	1.3 (0.3)	1.6 (0.4)	<0.001
Triglycerides (mmol/l)	1.4 (0.7)	1.3 (0.6)	0.670
fB-glucose (mmol/l)	5.1 (0.8)	5.0 (0.7)	0.421
2 h glucose (mmol/l)	6.8 (2.3)	6.9 (1.7)	0.170
Hemoglobin A1C (%)	5.5 (0.6)	5.4 (0.5)	0.228
Fasting-insulin (pmol/l)	11.8 (6.5)	9.9 (4.2)	0.083
HOMA-IR	1.6 (0.8)	1.3 (0.6)	<0.001
MS (NCEP) (%)	47 (36.4%)	57 (30.6%)	0.283
MS (IDF) (%)	54 (41.9%)	88 (47.3%)	0.340
PreDM (%)	36 (28.3%)	46 (25.3%)	0.548
T2D (%)	22 (17.3%)	18 (9.9%)	0.056
Smokers	22 (17.2%)	24 (13.0%)	0.310
Alcohol (mean g/l/day)	1.2 (0.3–3.3)	0.3 (0.0–1.6)	<0.001
Inactivity	33 (25.6%)	44 (23.7)	0.696

Data are means and Standard Deviation (SD); for the analysis data are log-transformed. Abbreviations: BMI indicates body mass index, BP, blood pressure; HOMA-IR, homeostasis model assessment of insulin resistance; Metabolic syndrome (MS) defined by National Cholesterol Education Panel (NCEP); Metabolic syndrome (MS) defined by International Diabetes Federation (IDF); PreDM, prediabetes by the ADA 2010 definition; T2D, type 2 diabetes.

The baseline levels of inflammatory markers in subjects with PreDM, T2D and NGT are compared in Table 2. The inflammatory cytokines IP-10 (251 pg/ml vs 209 pg/ml p = 0.043), TNF-α (10.4 pg/ml vs 6.9 pg/ml p = 0.020), RANTES (43.3 pg/ml vs 33.1 pg/ml p = 0.007), CD40L (3708 pg/ml vs 1671 pg/ml p = 0.002) and VEGF (269 pg/ml vs 174 pg/ml p = 0.013) levels were significantly higher in PreDM than in the NGT subjects. All these inflammatory markers, including IL-4 (10.4 pg/ml vs 5.9 pg/ml p = 0.041) remained significant after adjusting for the waist circumference. There was no statistical significant difference in the inflammatory makers between T2D and NGT even though the levels are remained increased. In contrast, the anti-inflammatory adipokine adiponectin levels were significantly lower in T2D in comparison with NGT subjects (24.9 ng/ml vs 36.2 ng/ml p = 0.043), however with the waist circumference adjustment the significance was abolished (p = 0.424).

**Table 2:** Levels of inflammatory markers in subjects with normoglycemica (NGT), prediabetes (PreDM) (ADA definition) and type 2 diabetes (T2D).

*Analyses based on log-trasformed values	NGT	Pre DM	T2DM	ANOVA			Adjusted for waist circumference^§^		
[pg/ml] Except for DPPIV and Adiponectin [ng/ml]				NGT vs Pre DM	NGT vs T2DM	Pre DM vs. T2DM	NGT vs Pre DM	NGT vs T2DM	Pre DM vs. T2DM
IL-1α*	32.2 ± 87.2	92.0 ± 427	48.7 ± 123	0.055	0.687	0.338	0.078	0.896	0.301
IL-1β*	3.4 ± 16.3	4.7 ± 15.6	2.0 ± 3.7	0.528	0.591	0.359	0.504	0.764	0.472
IL-1ra*	17.8 ± 64	42.7 ± 130	50.9 ± 198	0.086	0.083	0.697	0.109	0.066	0.500
IL-4*	5.9 ± 14.6	14.9 ± 63.7	10.4 ± 25.9	0.059	0.476	0.512	**0.041**	0.244	0.802
IL-6*	7.6 ± 29.1	11.3 ± 26.9	9.6 ± 20.0	0.303	0.677	0.741	0.474	0.785	0.830
IL-8*	15.5 ± 28.6	20.5 ± 26.0	18.9 ± 21.7	0.160	0.466	0.759	0.322	0.575	0.901
IL-17*	20.1 ± 67.8	40.8 ± 120	23.2 ± 37.8	0.058	0.831	0.267	0.083	0.815	0.359
IP-10*	209 ± 140	251 ± 179	254 ± 187	**0.043**	0.099	0.919	**0.050**	0.457	0.555
TNF-α*	6.9 ± 5.2	10.4 ± 18.0	8.5 ± 13.0	**0.020**	0.420	0.385	**0.027**	0.389	0.515
MCP-1*	594 ± 276	596 ± 275	583 ± 366	0.945	0.836	0.815	0.981	0.693	0.700
Active PAI-1*	73.1 ± 47.6	74.5 ± 40.4	72.8 ± 41.6	0.831	0.971	0.859	0.911	0.756	0.719
RANTES*	33.1 ± 29.3	43.3 ± 26.0	41.2 ± 28.7	**0.007**	0.105	0.702	**0.009**	0.108	0.829
MPO*	133 ± 107	153 ± 117	145 ± 147	0.183	0.543	0.713	0.228	0.422	0.968
CD40L*	1671 ± 4310	3708 ± 6016	3239 ± 4915	**0.002**	0.067	0.621	**0.010**	0.158	0.711
E-selectin*	34.2 ± 32.8	31.4 ± 14.6	35.2 ± 17.8	0.442	0.845	0.481	0.370	0.928	0.501
VCAM-1*	1547 ± 445	1481 ± 404	1554 ± 414	0.252	0.918	0.379	0.310	0.967	0.480
ICAM-1*	156 ± 79	146 ± 52	148 ± 52	0.284	0.529	0.866	0.317	0.488	0.994
VEGF*	174 ± 230	269 ± 411	183 ± 215	**0.013**	0.870	0.120	**0.015**	0.640	0.243
DPPIV*	491 ± 128	492 ± 167	534 ± 156	0.948	0.086	0.131	0.820	0.067	0.117
Adiponectin*	36.2 ± 34.5	28.3 ± 22.8	24.9 ± 18.4	0.060	0.043	0.570	0.245	0.424	0.999

Data are means (±SD) and *analyses based on log-transformed values. ^§^Adjusted for waist circumference. Gray highlighted P-values stay significant after Benjamini-Hochberg correction after multiple testing. Abbreviations: IL-1α, Interleukin 1α; IL-1β, Interleukin 1β, IL-1ra, Interleukin 1 receptor antagonist; IL-4, Interleukin 4; IL-6, Interleukin 6; IL-8, Interleukin 8; IL-17, Interleukin 17; IP-10, Interferon gamma-induced protein 10; TNF-α, Tumor necrosis factor α; MCP-1, Monocyte chemoattractant protein-1; active PAI-1, plasminogen activators inhibitor type 1 – activity; RANTES, Regulated on activation, normal T-cell and expressed and secreted; MPO, Myeloperoxidase; CD40L, Ligand for CD40; ICAM-1, Intercellular Adhesion Molecule 1; VCAM-1, Vascular Cell Adhesion Molecule 1; VEGF, Vascular endothelial growth factor; DPPIV, Dipeptidyl peptidase IV; NGT, subjects with normoglycemia; PreDM, prediabetes by the ADA definition; T2D, type 2 diabetes.

**Figure 2:**
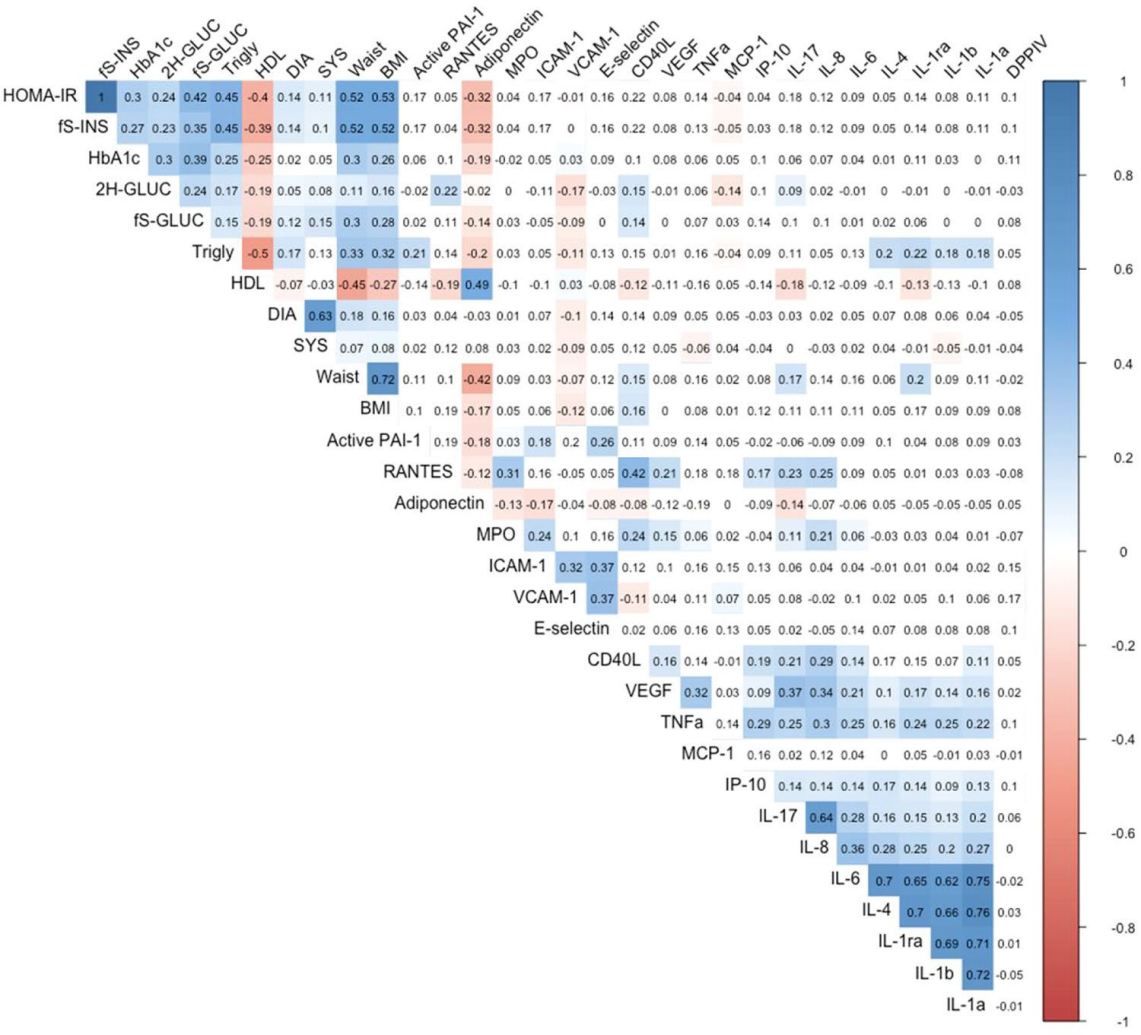
Spearman correlation coefficients between inflammatory markers, diabetes and cardiovascular risk factors. Red or blue color indicates the respective positive or negative correlation coefficient value intensities. Indices: BMI, Body mass index; DIA, Diastolic blood pressure; SYS, Systolic blood pressure, 2 H GLUC, 2 hour glucose; fS-GLUC, Fasting glucose, fS-INS, Fasting insulin; HbA1c, Hemoglobin A1c; HOMA-IR, Homeostasis model assessment for insulin resistance; HDL, High density lipoprotein; Trigly, Triglycerides; Waist, Waist circumference; IL-1α, Interleukin 1α; IL-1β, Interleukin 1β, IL-1ra, Interleukin 1 receptor antagonist; IL-4, Interleukin 4; IL-6, Interleukin 6; IL-8, Interleukin 8; IL-17, Interleukin 17; IP-10, Interferon gamma-induced protein 10; TNF-α, Tumor necrosis factor α; MCP-1, Monocyte chemoattractant protein-1; active PAI-1, plasminogen activators inhibitor type 1 – activity; RANTES, Regulated on activation, normal T-cell and expressed and secreted; MPO, Myeloperoxidase; CD40L, Ligand for CD40; ICAM-1, Intercellular Adhesion Molecule 1; VCAM-1, Vascular Cell Adhesion Molecule 1; VEGF, Vascular endothelial growth factor; DPPIV, Dipeptidyl peptidase IV. Units please see Table 2.

Correlations of IL-1α, IL-1β, IL-1Ra, IL-4, IL-6, IL-8, IL-17, IP-10, TNF-α, MCP1, active PAI-1, RANTES, CD40L, E-selectin, VCAM-1, ICAM-1, DPPIV and adiponectin with HOMA-IR and cardiovascular risk markers are shown in Fig. 2. Inflammatory markers IL-1Ra (r = 0.202; r = 0.172), IL-6 (r = 0.165; r = 0.112), IL-8 (r = 0.141; r = 0.112), CD40L (r = 0.148; r = 0.157), were positively correlated with waist circumference and BMI respectively. IL-17 (r = 0.171), TNF-α (r = 0.157), and E-selectin (r = 0.121) were found to be significantly positively correlated only with waist circumference and IP-10 (r = 0.117) and RANTES (r = 0.187) only with BMI.

In particular, CD40L had a positive correlation with fasting insulin, fasting glucose, 2 h glucose and HOMA-IR (r = 0.222; r = 0.142; r = 0.153; r = 0.222). Other significant correlations of IL-1Ra, IL-8, IL-17, TNF-α, active PAI-1, E-selectin, ICAM-1 were found with fasting insulin (r = 0.144; r = 0.117; r = 0.175; r = 0.131; r = 0.168; r = 0.162; r = 0.172; respectively) and HOMA-IR (r = 0.145; r = 0.122; r = 0.177; r = 0.136; r = 0.169; r = 0.157; r = 0.167; respectively). IP-10 correlated with fasting glucose (r = 0.137) and RANTES correlated with 2 h glucose (r = 0.222) together with the CD40L. The novel adipokine DPPIV was positively correlated with HOMA-IR (r = 0.105) and HbA1c (r = 0.112).

Inflammatory markers correlated also with lipids: IL-1α, IL-1β, IL-1Ra, IL-4, IL-6, TNF-α, active PAI-1, RANTES, CD40L, and E-selectin had the strongest positive correlations with triglycerides (r = 0.187; r = 0.180; r = 0.227; r = 0.207; r = 0.132; r = 0.163; r = 0.212; r = 0.141; r = 0.152; r = 0.131; respectively). On the contrary, the cytokines IL-1β, IL-1Ra, IL-8, IL-17, IP-10, TNF-α, active PAI-1, RANTES, CD40L, and ICAM-1 had a significant negative correlation with HDL cholesterol (r = −0.137; r = −0.137; r = −0.118; r = −0.176; r = −0.137; r = −0.166; r = −0.142; r = −0.192; r = −0.122; r = −0.112; respectively). In addition, only RANTES and CD40L had a significant positive correlation with systolic blood pressure (r = 0.121; r = 0.121, respectively). Furthermore, CD40L and E-selectin correlated positively with diastolic blood pressure (r = 0.142; r = 0.138, respectively).

The anti-inflammatory adipokine adiponectin had a significant negative correlation with BMI, waist circumference, triglycerides and glucose markers such as fasting glucose, fasting insulin, HOMA-IR and HbA1c (r = − 0.168; r = − 0.409; r = − 0.203; r = − 0.143; r = − 0.322; r = − 0.329; r = − 0.201; respectively), while adiponectin was strongly associated with HDL cholesterol levels (r = 0.496).

**Figure 3:**
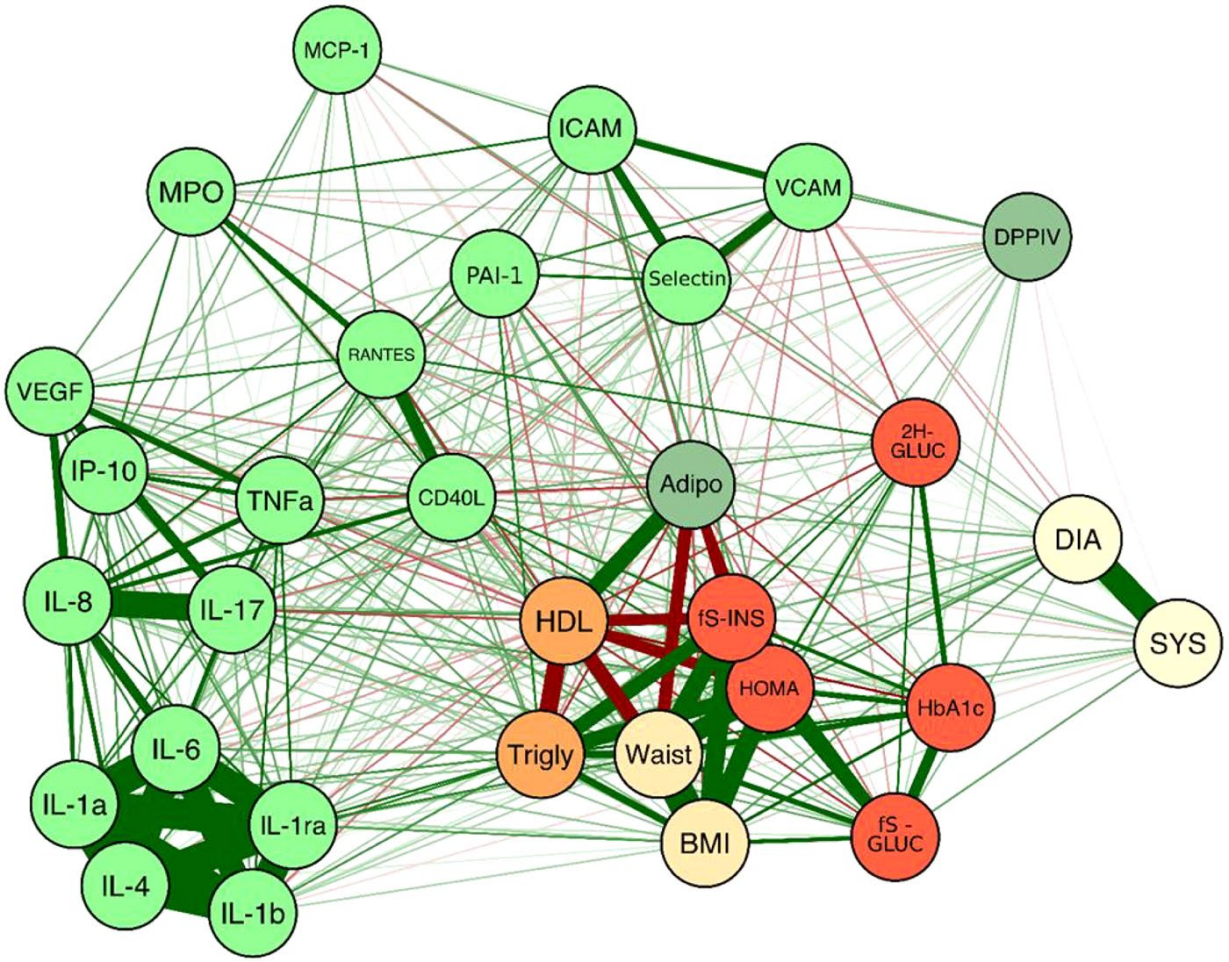
Correlation Network. The thickness of the lines represent the strength of the correlation. Green lines – positive correlation. Red lines – negative correlation. There are strong positive correlations between BMI, waist circumference, HOMA-IR, systolic and diastolic blood pressure. In the cytokines, there are four positive correlated clusters: (1) IL-1α, IL-1β, IL-1Ra, IL-4, and IL-6; (2) VEGF, IP-10, TNF-α, IL-8 and IL-17; (3) MPO, RANTES and CD40L; (4) E-selectin, ICAM-1 and VCAM-1. Adiponectin and HDL cholesterol are positively correlated while HDL is negative with fasting insulin (fS-INS), waist circumference and triglycerides. BMI, Body mass index; DIA, Diastolic pressure; SYS, Systolic blood pressure, 2 H GLUC, 2 hour glucose; fS-GLUC, Fasting glucose, fS-INS, Fasting insulin; HbA1c, Hemoglobin A1c; HOMA, Homeostasis model assessment for insulin resistance; HDL, High density lipoprotein; Trigly, Triglycerides; Waist, Waist circumference; IL-1α, Interleukin 1α; IL-1α, Interleukin 1β, IL-1ra, Interleukin 1 receptor antagonist; IL-4, Interleukin 4; IL-6, Interleukin 6; IL-8, Interleukin 8; IL-17, Interleukin 17; IP-10, Interferon gamma-induced protein 10; TNF-α, Tumor necrosis factor α; MCP-1, Monocyte chemoattractant protein-1; active PAI-1, plasminogen activators inhibitor type 1 – activity; RANTES, Regulated on activation, normal T-cell and expressed and secreted; MPO, Myeloperoxidase; CD40L, Ligand for CD40; ICAM-1, Intercellular Adhesion Molecule 1; VCAM-1, Vascular Cell Adhesion Molecule 1; VEGF, Vascular endothelial growth factor; DPPIV, Dipeptidyl peptidase IV.

A summary of the different parameters and their correlations is presented in Fig. 3.

## Discussion

In our cross sectional study of elderly subjects we observed increased IL-1α, IL-1Ra, IL-4, IL-6, IL-8, IL-17, TNF-α, IP-10, RANTES, CD40L and VEGF levels already in the individuals with prediabetes, while MCP-1, active PAI-1, MPO, E-selectin, VCAM-1, ICAM-1 and DPPIV were unchanged, and adiponectin levels decreased.

Many of the inflammatory markers are associated with obesity^24,25^. Accordingly, in our study IL-1Ra, IL-6, IL-8, and CD-40L levels correlated with both waist circumference and BMI. IL-17, TNF-α, and E-selectin correlated only with waist circumference and IP-10, RANTES and VCAM-1 only with BMI. In addition, the markers significantly associate with established cardiovascular risk factors such as HOMA-IR, high blood pressure, high triglycerides, and low HDL cholesterol (Fig. 2).

After the adjustment for waist circumference the significance persistent for IL-4, IP-10, TNF-α, RANTES, CD40L and VEGF, indicating that inflammation is an independent hallmark in prediabetes.

Inflammation has been considered a hallmark in the pathogenesis of T2D with an activation of the innate immunity in adipose tissue^3,26^. Chronic adipose tissue inflammation can lead to insulin resistance^8^ and endothelial dysfunction^5^.

TNF-α is secreted from white visceral adipose tissue (WAT), which is infiltrated with macrophages, locally regulating inflammation^27^. In agreement with previous studies, waist circumference was positively associated with elevated TNF-α levels in prediabetic subjects^28^ and insulin resistance^29^.

IP-10 and RANTES are produced by the adipose tissue and the associated macrophages^30^ and increased in atherosclerosis and coronary artery disease^1,31^. We found significant elevation of IP-10 in prediabetic subjects, which is consistent with previous results of a cross sectional study of diabetic subjects with non-alcoholic fatty liver disease (NAFLD) compared to non-diabetic NAFLD subjects^32^. IP-10 levels were not significantly different in either IGT or T2D subjects in the KORA study, a population-based study consisting of 236 subjects with T2D, 242 subjects with IGT and 244 normoglycemic control subjects^24^. Their number of diabetic subjects was higher compared to our study. Furthermore, in our study prediabetes was defined by the ADA definition using IFG, IGT, as well as elevated HbA1c, in comparison to the WHO IGT definition used in the KORA study. In the KORA study, RANTES levels were higher in diabetic subjects and in subjects with IGT^24^. Furthermore, adjustments for classic metabolic risk factors did not alter these findings. In our subjects, RANTES was increased in both prediabetic and diabetic subjects, suggesting that RANTES might be a useful marker in predicting the early risks of CVD in these subjects^1^. In line with this, inhibition of endogenous RANTES has been shown to diminish leukocyte recruitment and reduce progression of an established atherosclerosis model, using LDLr−/− C57BL/6 mice^33^. Furthermore, Canoui-Poitrine *et al*. found in a 10-year follow up study of 9771 men that RANTES was associated with ischemic stroke, independently of the traditional cardiovascular risk markers. The authors suggested that RANTES in combination with IP-10 might improve the prediction of ischemic stroke^34^.

CD40L, is a transmembrane protein receptor ligand and a member of the TNF superfamily, and has been associated with the pathophysiology of atherosclerosis and regarded as biomarker for risk of CVD^5^. We have previously shown elevated CD40L levels in IGT subjects^35^.

The correlations of our study are demonstrated in Fig. 3: Clusters 1, consisting of IL-1α, IL-1β, IL-1Ra, IL-4, and IL-6, demonstrates activation of macrophages, T cells, endothelial cells and fibroblasts (Fig. 3). There is a strong association within the cytokines, but weaker correlations with glucose or BMI (Fig. 2). This might be explained by the relative good health status of our subjects, which were overweight, but not obese, with a high number of prediabetic subjects, but not with T2D. Cluster 2 (VEGF, IP-10, TNF-α, IL-8 and IL-17) represents activation of effector T cells, macrophages, endothelial cells and fibroblasts. Cluster 3 (MPO, RANTES and CD40L) demonstrates activation of neutrophils and T cells, while cluster 4 (E-selectin, ICAM-1 and VCAM-1) correlates to makers of endothelial activation, lymphocytes, and dendritic cells.

In addition to cytokines, the adipose tissue secretes a number of other substances called adipokines, e.g. DPPIV and adiponectin. Lee *et al*. showed that circulating DPPIV levels were higher in the T2D subjects^36^. Similarly, in our study we found elevated DPPIV levels in diabetic subjects, although they were not statistically significant in comparison to NGT subjects. In addition, the DPPIV levels were significantly correlated with the markers of glucose metabolism HOMA-IR and Hb1c as previous reported by Lamers *et al*.^16^.

The anti-inflammatory adipokine adiponectin was significantly reduced in T2D subjects and highly correlated with other confounding risk factors of T2D and CVD. These results are in accordance with our previous studies and strongly supports the association between markers of endothelial dysfunction and inflammatory markers^37^. Ruotsalainen *et al*. concluded that low-grade inflammation markers may precede the elevation of adhesion molecules levels like E-selectin, ICAM-1 and VCAM-1 (Cluster 4)^38^.

Our subjects were 62.1 years of age. There is an age-associated decrease in β-adrenergic receptors^39^ and this reduction could have an effect on insulin secretion^40^. The decrease in the age related adrenergic responsiveness combined with the insulin resistance and elevated inflammatory markers in the elderly subjects might contribute to the progression of CVD.

The strength of our study is an unselected elderly population with a similar background and lifestyle using the definition of prediabetes including HbA1c in studying inflammatory mediators. However, limited to a subcohort of the entire study, we had reduced statistical power to compare the differences between different subgroups and a low number of T2D subjects (IFG, IGT and HbA1c).

Our participants were middle-aged Caucasians and whether or not our findings also apply to other ethnic and age groups is unknown. In terms of bias and variability, we evaluated 129 men and 186 women in Northern Finland with the potential confounding factors like BMI, HOMA-IR, high blood pressure, high triglycerides, and low HDL-cholesterol. More measurements would have reduced the amount of the random variation, but our study was designed as a cross section study.

In conclusion, low-grade inflammation measured with IP-10, TNF-α, RANTES, CD40L and VEGF was associated with prediabetes as defined by both OGTT and/or high HbA1c, with comparable levels of inflammation in subjects with prediabetes and T2D. Low-grade inflammation is one of the factors underlying the association between intermediate hyperglycemia and increased risk factors for cardiovascular diseases, evident already in prediabetic subjects. Prospective studies are needed to evaluate the role of the inflammation in prediabetic subjects, and the role of RANTES, CD40L as a predictor of subsequent T2D and CVD.
